# Role of Psychosocial Factors and Health Literacy in Pregnant Women’s Intention to Use a Decision Aid for Down Syndrome Screening: A Theory-Based Web Survey

**DOI:** 10.2196/jmir.6362

**Published:** 2016-10-28

**Authors:** Agathe Delanoë, Johanie Lépine, Stéphane Turcotte, Maria Esther Leiva Portocarrero, Hubert Robitaille, Anik MC Giguère, Brenda J Wilson, Holly O Witteman, Isabelle Lévesque, Laurence Guillaumie, France Légaré

**Affiliations:** ^1^ Populations Health and Optimal Health Practices Research Group CHU de Québec-Université Laval Quebec City, QC Canada; ^2^ Quebec Centre of Excellence on Aging CHU de Québec-Université Laval Quebec City, QC Canada; ^3^ Office of Education and Continuing Professional Development Faculty of Medicine Université Laval Quebec City, QC Canada; ^4^ School of Epidemiology Public Health and Preventive Medicine University of Ottawa Ottawa, ON Canada; ^5^ Department of Family Medicine and Emergency Medicine Faculty of Medicine Université Laval Quebec City, QC Canada; ^6^ Department of Obstetrics and Gynecology Faculty of Medicine Université Laval Quebec City, QC Canada; ^7^ Tier 1 Canada Research Chair in Shared Decision Making and Knowledge Translation Faculty of Medicine Université Laval Quebec City, QC Canada

**Keywords:** decision aids, behavior, intention, prenatal diagnosis, decision making, health literacy

## Abstract

**Background:**

Deciding about undergoing prenatal screening is difficult, as it entails risks, potential loss and regrets, and challenges to personal values. Shared decision making and decision aids (DAs) can help pregnant women give informed and values-based consent or refusal to prenatal screening, but little is known about factors influencing the use of DAs.

**Objective:**

The objective of this study was to identify the influence of psychosocial factors on pregnant women’s intention to use a DA for prenatal screening for Down syndrome (DS). We also added health literacy variables to explore their influence on pregnant women’s intention.

**Methods:**

We conducted a survey of pregnant women in the province of Quebec (Canada) using a Web panel. Eligibility criteria included age >18 years, >16 weeks pregnant, low-risk pregnancy, and having decided about prenatal screening for the current pregnancy. We collected data based on an extended version of the Theory of Planned Behavior assessing 7 psychosocial constructs (intention, attitude, anticipated regret, subjective norm, descriptive norm, moral norm, and perceived control), 3 related sets of beliefs (behavioral, normative, and control beliefs), 4 health literacy variables, and sociodemographics. Eligible women watched a video depicting the behavior of interest before completing a Web-based questionnaire. We performed descriptive, bivariate, and ordinal logistic regression analyses.

**Results:**

Of the 383 eligible pregnant women who agreed to participate, 350 pregnant women completed the Web-based questionnaire and 346 were retained for analysis (completion rate 350/383, 91.4%; mean age 30.1, SD 4.3, years). In order of importance, factors influencing intention to use a DA for prenatal screening for DS were attitude (odds ratio, OR, 9.16, 95% CI 4.02-20.85), moral norm (OR 7.97, 95% CI 4.49-14.14), descriptive norm (OR 2.83, 95% CI 1.63-4.92), and anticipated regret (OR 2.43, 95% CI 1.71-3.46). Specific attitudinal beliefs significantly related to intention were that using a DA would reassure them (OR 2.55, 95% CI 1.73-4.01), facilitate their reflections with their spouse (OR 1.55, 95% CI 1.05-2.29), and let them know about the advantages of doing or not doing the test (OR 1.53, 95% CI 1.05-2.24). Health literacy did not add to the predictive power of our model (P values range .43-.92).

**Conclusions:**

Implementation interventions targeting the use of a DA for prenatal screening for DS by pregnant women should address a number of modifiable factors, especially by introducing the advantages of using the DA (attitude), informing pregnant women that they might regret not using it (anticipated regret), and presenting the use of DAs as a common practice (descriptive norm). However, interventions on moral norms related to the use of DA should be treated with caution. Further studies that include populations with low health literacy are needed before decisive claims can be made.

## Introduction

Prenatal tests for Down syndrome have become routine in many developed countries through population-based screening programs [1]. For women, their partners, and clinicians, the decision about whether or not to do the tests can be a difficult one to make [2-4]. The initial decision about screening may seem banal, but it can be the first of a series of increasingly difficult and sensitive decisions. First, although screening results may decrease women’s uncertainty, there is still a risk of false-positive or false-negative results. Second, if the results are positive, women are faced with a further decision about amniocentesis, a more invasive test that carries the risk of losing the fetus. Finally, if the results of the amniocentesis are positive, the woman has to decide whether to have an abortion or to prepare for a child with special needs. Thus, each successive decision entails more physically invasive procedures, more significant challenges to women’s personal values, and changes in their hopes for the future.

Although more accurate screening tests providing earlier results are increasingly available, such as the new noninvasive prenatal test (NIPT) [5], decisions about prenatal screening still gain in complexity [6-8]. In this rapidly evolving clinical context, several decisional needs are still unmet and new ones are emerging that urgently need to be addressed [5,9,10].

Patient decision aids (DAs) are decision support tools that could help women and their partners to make informed prenatal screening decisions congruent with their values. An informed choice is one in which a patient has understood the evidence related to each option as well as considered what best fits his or her values and preferences and made a decision consistent with this [11]. DAs are therefore designed to help patients to engage in decision making not only by providing best evidence on the options, but also by helping them clarify and communicate what is most important to them about the decision (values and preferences) [12]. DAs have been found to stimulate people to take a more active role in decision making, to increase knowledge, to improve the accuracy of risk perception, to improve congruence between choice and patient values, and to decrease decisional conflict (personal uncertainty) as well as decision regret [12]. Providing detailed information on prenatal testing has been shown to be significantly associated with an increase in patient satisfaction [13] and DAs have been shown to decrease anxiety [14]. Although several DAs are available for prenatal screening, they are not routinely implemented [4,15], and none meet all the International Patient Decision Aids Standards criteria, as our earlier scan has demonstrated [16]. DAs have not yet, in fact, been routinely implemented in many clinical contexts [17]. This has been attributed to health professionals’ lack of training in using them, their lack of trust in their content or their disagreement with it, or their belief that patients facing a difficult diagnosis do not want to take responsibility for decisions [17]. The successful implementation of DAs is likely to be affected by a number of factors [18]. A recent study suggested that the main factors influencing health professionals’ use of a DA in prenatal care were their positive impression of the DA, its availability in their offices, and their colleagues’ approval of its use [19]. Another study showed that, for pregnant women, the main factors were their partner’s opinion, the DA being explained by and discussed with the health professional, and whether or not the women had ever encountered a DA before (Leiva Portocarrero, M. Sc., personal written communication, February 2016).

In recent years, adoption of new health-related behaviors, including those needed to help disseminate DAs effectively, has been studied with the help of behavior change theories [20-22]. These theories allow identification of the modifiable factors influencing behavior adoption that should be targeted in implementation interventions in order to produce the needed behavior change [23,24]. Most of these behavior change theories rely on the assessment of the determinants of behavioral intention, which is considered to be the best predictor of behavior adoption [25]. More specifically, the use of a behavior change theory could better enable the identification of a set of behavioral factors influencing pregnant women’s intention to use a DA, which could then help in designing an effective implementation intervention.

In addition, in the context of prenatal screening, pregnant women with fewer years of education have reported being less willing to engage in shared decision making (SDM) [26]. This is congruent with a growing body of literature indicating that health literacy is a potential barrier to SDM [27-29] and to the use of DAs [30,31]. Health literacy includes self-confidence, social skills, and social networks as well as literacy and numeracy [32-35], and all these dimensions are likely to affect patients’ intention to use a DA [30]. Studies have also demonstrated that health literacy influences patients’ motivation to manage their health [36,37] and their attitude toward SDM, especially their desire for involvement in the decision [31,38-44] and for information [45,46], their perception of decisional responsibility [47], their perception of the harms and benefits of treatments [33,48-52], and their capacity to understand genetic information [53] and laboratory test results [54]. Research has also shown that lower health literacy levels among pregnant women are associated with poorer understanding of prenatal screening tests [55].

Measuring how much an enriched set of factors influences the uptake of DAs by patients could inform the design of theory-based interventions to support their implementation in the clinic [56]. Therefore, the aim of this study was to identify the factors influencing pregnant women’s intention to use a DA about prenatal screening for Down syndrome. More specifically, the objectives were the following: (1) based on an extended model of behavior change [57], to identify the psychosocial determinants influencing pregnant women’s intention to use a DA about prenatal screening for Down syndrome; (2) to explore adding health literacy as a direct determinant of intention or as a variable that could moderate the influence of other direct determinants of intention.

## Methods

### Study Design

This cross-sectional Web-based survey was embedded in a large Canadian research initiative called the PEGASUS project (Personalized Genomics for Prenatal Aneuploidy Screening Using Maternal Blood) aiming to validate the performance and utility of the NIPT in the general population. In this large initiative, our overarching aim was to inform the future implementation of a DA to foster SDM in the context of prenatal screening for Down syndrome. Ethics approval was obtained from the research ethics boards of the Centre intégré universitaire de santé et de services sociaux de la Capitale-Nationale (#2013-2014-29), the Centre intégré de santé et de services sociaux de Chaudière-Appalaches (CER-1415-910), and the CHU de Québec (#B14-02-1929). We used the CHERRIES (Checklist for Reporting Results of Internet E-Surveys) checklist to guide the reporting of our Web-based survey [58].

### Theoretical Framework

**Figure 1:**
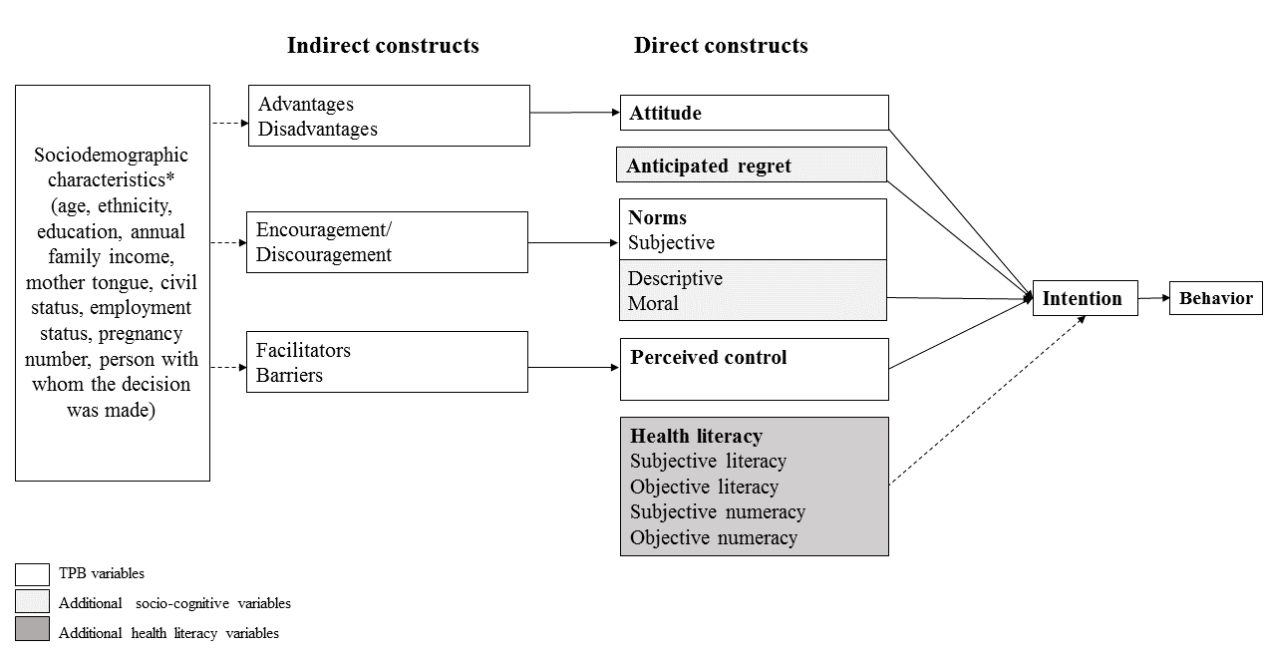
Extended model of behavior change. The constructs take into account influence of sociodemographic characteristics*. TPB: Theory of Planned Behavior.

The theoretical framework of this study was the Theory of Planned Behavior (TPB), which is one of the highest-performing and commonly used theories for identifying the determinants of intention [59]. According to the TPB, the direct determinants of intention are attitude (perceived advantages of adopting the behavior), subjective norm (the perceived social pressure from significant others to perform the behavior), and perceived behavioral control (perceived control over performing the targeted behavior). These direct determinants of intention are respectively associated with indirect constructs: (1) attitudinal beliefs (perceived advantages and disadvantages of using a DA for prenatal screening for Down syndrome during the course of a prenatal care visit during a subsequent pregnancy); (2) normative beliefs (a woman’s perceptions of to what extent partner, parents, or friends want her to perform the behavior); and (3) control beliefs (perceived barriers and facilitators of engaging in the behavior) [25]. The identification of beliefs that are associated with the intention to perform the behavior allows the specification of precise targets in future interventions. The extended behavior change model used in this study includes the main determinants of intention according to the TPB (attitude, subjective norm, and perceived control), as well as additional constructs known to improve the predictive capacity of the TPB: (1) anticipated regret, or an estimation of the regret that would result from not adopting the behavior; (2) descriptive norm, or the perceived prevalence of the practice; and (3) moral norm, or the moral principles involved [60-63] (see Figure 1).

### Participants and Recruitment

From September 16, 2015, to October 8, 2015, we recruited eligible pregnant women (Table 1). Eligibility criteria included the following: being at least 18 years old, not less than 16 weeks pregnant, not presenting a high-risk pregnancy (eg, preeclampsia, gestational diabetes, multiple pregnancy), and having already decided about prenatal screening for the current pregnancy. Women who participated in a previous phase of the research were excluded. A private company specialized in polling was mandated to recruit eligible pregnant women in the province of Quebec (Canada) using a Web panel of willing participants in Internet surveys. Canada’s health care system consists of 13 (10 provincial and 3 territorial) independent health care systems. In this study, we focused on the province of Quebec, which is the second most populous Canadian province. First, the survey company sent an email invitation to all women on the panel aged from 18 to 44 years. After 2 weeks, to enhance recruitment, the same email invitation was sent to men on the panel aged from 25 to 44 years, as in this age range their partner was more likely to meet our eligibility criteria. The email invitation included the following information: (1) Subject: Research in health services led by Université Laval; (2) Financial compensation: 25 Canadian dollars; (3) Time to answer: next 5 business days; and (4) Personalized link to survey. Nonrespondents received a reminder every 2 weeks until the survey was closed. All interested persons who clicked on the personalized survey link were directed to the closed survey (password-protected) and asked to answer preliminary eligibility questions. Special filters allowed selection of the female partners of recruited men on the panel. Once eligibility criteria were confirmed, eligible women started the voluntary survey.

### Data Collection

Participating pregnant women completed the Web-based survey through 39 Web pages that included up to 7 items, always appearing in the same order (see Multimedia Appendix 1). Clear preliminary statements provided information about the study and instructions and allowed participants to confirm their consent. To foster participation, the survey enabled pregnant women to stop the survey at any time and to restart it as long as the personalized survey link was active. No completeness check was possible before submitting the questionnaire. Once an item was answered, the answer could not be changed, as many items were similar and we wanted to test if participants’ responses were consistent. Once data collection was completed, the contracted company sent us the data anonymously, which were then stored on our secure network (password-protected).

In earlier research on factors that influence health-related behavior change [64,65], we observed that it is helpful to give participants a vicarious experience of the behavior of interest in order for them to understand it better [66]. As the pregnant women were not expected to have experienced the use of a DA, to help them understand the behavior of interest (action: use; target: a DA for prenatal screening for Down syndrome; context: during the course of prenatal care visits during a subsequent pregnancy; time: not specified), we asked them to watch a 10-minute video first. The video depicted a prenatal care follow-up during which a pregnant woman, her partner, and a health professional used a DA to decide about prenatal screening for Down syndrome. Production of this video had followed a validated process and had proved successful for communicating the behavior of interest [67]. The DA is available in Multimedia Appendix 2. After watching the whole video, eligible women answered the Web-based questionnaire based on the TPB but which included additional psychosocial factors known to influence the uptake of a new behavior [57,60-63]. In a previous step of the project, we had conducted a pilot study to validate this questionnaire [68]. We also measured underlying salient beliefs related to the direct constructs as elicited in a previous qualitative study (Leiva Portocarrero, M. Sc., personal written communication, February 2016). Using 52 closed items scored on a 5-point Likert-type scale, we measured intention, attitude, subjective norm, perceived behavioral control, anticipated regret, descriptive norm, moral norm, attitudinal beliefs, normative beliefs, and control beliefs. Except for attitude and anticipated regret, all direct constructs were assessed with multi-item measures. Anticipated regret was measured with 2 items and attitude with 6 items using bipolar adjective pairs assessing cognitive and affective dimensions of women’s attitudes. Cronbach alphas indicated good reliability of multi-items measuring each construct (alpha range .67-.94, Table 2). The questionnaire was developed following Ajzen's guidelines [69] and referred to using a DA to decide about prenatal screening for Down syndrome. All in all, the questionnaire included 52 psychosocial items, 9 sociodemographic items, and 50 health literacy items, for a total of 111 items. It was available in French and English.

To assess health literacy, after consulting with experts in the field [30,48,54,70] and reviewing multiple systematic reviews [71-75], we chose to use both subjective and objective scales. While objective scales measure competencies, subjective scales measure the perception of competencies and have been shown to reduce burden of participants [76-78]. We thus assessed pregnant women’s levels of health literacy using the following 4 complementary scales that measure health literacy and numeracy both objectively and subjectively: (1) the short version of the Test of Functional Health Literacy in Adults (S-TOFHLA), the literacy part only, which comprises 36 blank spaces and 4 choices of words to fill the blanks [79,80]; (2) a total of 3 self-administered health literacy questions (3HLQ, 5-point Likert scale: range 0 to 4, final score range 0 to 12) [81]; (3) a total of 3 numeracy questions (3NQ, 3 items, correct answers range 0 to 3) [82]; and (4) the Subjective Numeracy Scale (SNS, 2 subscales of 4 closed questions scored on a 5-point Likert scale, mean score range 1 to 5 for both subscales and complete scale) [76]. Finally, we assessed sociodemographics such as age, clinician in charge of monitoring, mother tongue, ethnicity, civil status, employment status, annual family income, education, and pregnancy number.

### Statistical Analysis

#### Sample Size

Informed by the test-retest of the questionnaire, we postulated that analysis in this study would be best performed using a logistic regression model. On the basis of Peduzzi and colleagues’ works on sample size [83] and taking into account all independent variables in this study, we found that a sample of 350 women was sufficient according to the principle of the number of events per variable, which asserts that a minimum of 10 events per variable is required to perform valid logistic regression models [83].

#### Data Analysis

First, we used simple descriptive statistics (means, standard deviations, medians, quartiles, and percentages) to summarize sociodemographic, sociocognitive, and health literacy variables. For each sociocognitive construct we verified internal consistency by calculating Cronbach alphas, except anticipated regret, for which we did a Spearman correlation. Intention was not normally distributed and, as it could not be transformed successfully, we created 3 categories of intention—scores < 4, scores=4, and scores >4—based on the fact that the subtle gradations that span the 5-point scale made each category distinct in the clinical sense. In line with earlier research on health literacy, we dichotomized all health literacy variables: scores of 3HLQ were dichotomized as inadequate (≤10) or adequate (>10) [52]; scores of 3NQ were dichotomized as < 3 versus 3 correct answers [84]; and scores of SNS were dichotomized at the median (<3.75 vs≥3.75). Scores of S-TOFHLA could not be further analyzed because the lack of variability in the distribution did not enable us to discriminate among the pregnant women’s scores. We performed bivariate ordinal logistic regression to measure difference in the distribution of all sociocognitive variables, all sociodemographic variables, and the 4 health literacy variables, according to each of the 3 intention categories. We then performed a first ordinal logistic regression in which only TPB variables were included. We used a backward approach to test the model adjustment with sociodemographic variables. Next, we compared the extended TPB model, including the additional variables of anticipated regret, descriptive norm, and moral norm, with the preceding model. We then added each health literacy variable to the extended model of regression, except for objective literacy (S-TOFHLA), which lacked variability. We also tested the interaction between health literacy variables and all direct constructs. Then, to identify significant underlying beliefs, we replaced significant constructs that determined women’s intention with their associated beliefs and performed the regression model with these significant factors (e.g., attitude was replaced by its underlying beliefs). Following a backward approach, we kept significant variables (*P*<.05). For all comparison models described above, we used deviance to compare the 2 nested models to identify which one was best.

## Results

### Flow of Participants and Participants’ Characteristics

**Figure 2:**
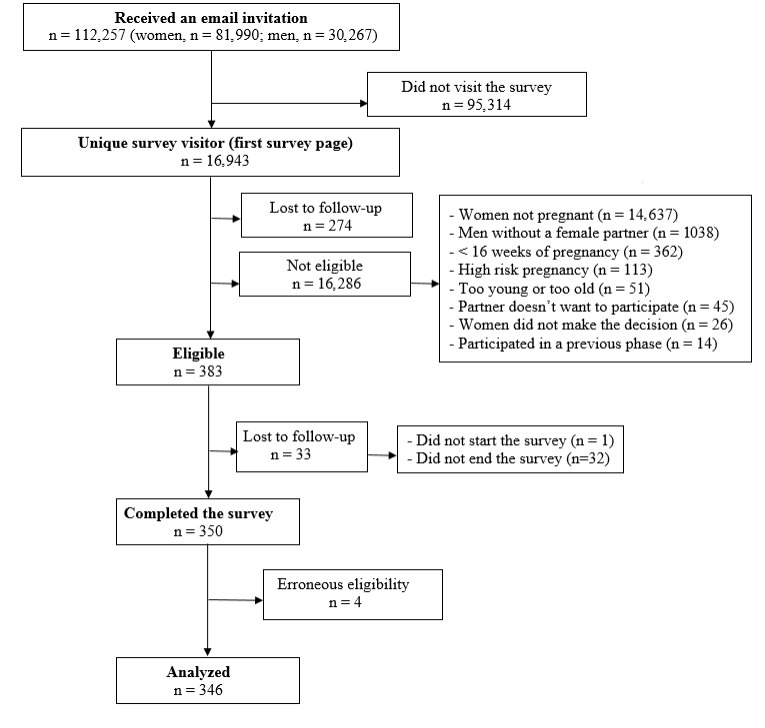
Flow of participants.

Details on flow of participants are depicted in Figure 2. On the basis of the CHERRIES statement [58], we considered all potentially eligible participants who clicked on the personalized link to visit the survey as unique survey visitors. As the Web survey did not include a middle stage between visiting the website and visiting the first survey page, we adapted the CHERRIES criteria to calculate the study's response rates [58]. Accordingly, view rate (ratio of unique survey visitors/unique receiver of survey invitation), participation rate (ratio of users who agreed to participate/unique survey visitors), and completion rate (ratio of users who finished the survey/users who agreed to participate) were respectively 15.09% (16,943/112,257), 2.26% (383/16,943), and 91.4% (350/383) (Figure 2).

Time of completion was not kept for analysis as participating women could stop and restart the survey. No data were missing as the Web-based questionnaire did not accept unanswered items. Mean age of pregnant women was 31 years. Out of 346 women retained for data analysis, 319 (92.2%) were white, 318 (91.9%) were French-speaking, and 168 (48.6%) had a university degree (see Table 1).

**Table 1:** Participant characteristics (n=346).

Characteristics		n (%)
Age, years, mean (SD)		30.1 (4.3)
**Monitored by**		
	Obstetrician-gynecologist	201 (58.1)
	Family physician	105 (30.3)
	Midwife	30 (8.7)
	Other	10 (2.9)
**Mother tongue**		
	French	318 (91.9)
	English	18 (5.2)
	Other	10 (2.9)
**Ethnicity**		
	White	319 (92.2)
	African or African American	4 (1.2)
	Latin American	5 (1.4)
	Arab	8 (2.2)
	Chinese	2 (0.6)
	Filipino	1 (0.3)
	Korean	1 (0.3)
	Other	6 (1.8)
**Civil status**		
	Single	23 (6.6)
	Not single	323 (93.4)
**Employment status**		
	Full time	269 (77.8)
	Part time	45 (13.0)
	Unemployed	23 (6.6)
	Student	9 (2.6)
**Annual family income^a^**		
	< $29,999	24 (6.9)
	$30,000-$59,999	74 (21.4)
	$60,000-$99,999	146 (42.2)
	>$100,000	82 (23.7)
	No answer	20 (5.8)
**Education**		
	No high school	4 (1.2)
	High school diploma	25 (7.2)
	Professional diploma	61 (17.6)
	Collegial diploma	88 (25.4)
	University degree	168 (48.6)
**Pregnancy number**		
	First	130 (37.6)
	Second	137 (39.6)
	Third	40 (11.5)
	Fourth or more	39 (11.3)
**Health literacy, median/total score (% higher level^b^** **)**		
	Objective literacy	36.00/36 (N/A^c^)
	Subjective literacy	10.00/12 (51.5)
	Objective numeracy	3.00/3 (56.7)
	Subjective numeracy (total)	3.88/5 (55.2)

^a^Canadian dollars.

^b^Higher level corresponds to the higher category of each scale when scores were dichotomized.

^c^N/A: not applicable; no further analyses were done for this scale because its lack of variability did not permit dichotomization of the scores.

**Table 2:** Intention and psychosocial factor analysis (n=346).

Construct^a^	Intention score, by category, median (Q1-Q3)			Cronbach alpha	*P* value^b^
	<4 n=109 (31.5%)	4 n=92 (26.6%)	>4 n=145 (41.9%)		
Attitude (6 items)	3.50 (2.33-3.67)	3.67 (3.00-4.00)	4.33 (4.00-4.67)	.85	< .001
Anticipated regret (2 items)	2.00 (1.50-3.00)	3.00 (2.50-3.50)	3.50 (3.00-4.00)	.67^c^	< .001
Subjective norm (3 items)	3.00 (3.00-3.67)	4.00 (3.67-4.33)	4.67 (4.33-5.00)	.84	< .001
Descriptive norm (3 items)	3.00 (2.67-4.00)	4.00 (3.33-4.00)	4.00 (4.00-4.67)	.85	< .001
Moral norm (3 items)	3.33 (3.00-3.67)	4.00 (3.83-4.00)	5.00 (4.67-5.00)	.90	< .001
Perceived control (5 items)	4.00 (3.50-4.25)	4.25 (3.75-4.50)	4.50 (4.25-5.00)	.67	< .001

^a^Range from 1 to 5.

^b^Bivariate ordinal logistic regression.

^c^Spearman correlation.

### Descriptive and Bivariate Analyses

Intention to use the DA for deciding about prenatal Down syndrome screening during a subsequent pregnancy, and factors of this intention, showed generally high scores. Among the 346 pregnant women, 109 (31.5%) had an intention score of < 4 out of 5, a total of 92 (26.6%) had an intention score of 4 out of 5, and 145 (41.9%) had an intention score of >4 out of 5. All the direct determinants of intention showed similar scores (≥ 3.00 out of 5, see Table 2 and Multimedia Appendix 3 for details), except anticipated regret, which showed a median score of 2.00. The level of health literacy was generally high. A median score of 36.00 out of 36 was obtained for objective literacy (S-TOFHLA), 10.00 out of 12 for subjective literacy (3HLQ), 3.00 out of 3 for objective numeracy (3NQ), and 3.88 out of 5 for subjective numeracy (SNS total score; Table 1).

With our bivariate analysis, we found that all sociocognitive factors were significantly associated with intention (*P*<.001 for all, Table 2).

No sociodemographic and health literacy variable was significantly associated with intention (Multimedia Appendices 4 and 5). In exploring correlations between health literacy variables and sociocognitive constructs, we found the most frequent association was with perceived control, which showed significant associations with all health literacy variables except objective numeracy (Multimedia Appendix 6). In addition, we observed that all health literacy scales were correlated among themselves (rho range .14-.89, *P* value range .007-.001; see Multimedia Appendix 6).

### Multivariate Analysis

Finally, we identified the most significant factors in women’s intention to use the DA. In the first multivariate model, including only TPB variables, attitude (odds ratio, OR, 13.38, 95% CI 6.40-27.90), subjective norm (OR 3.64, 95% CI 2.33-5.70), and perceived control (OR 2.36, 95% CI 1.43-3.90) were significant factors of pregnant women’s intention (Table 3). No sociodemographic variable was added to the model.

In the second multivariate model, still based on the TPB but including the additional variables of anticipated regret, descriptive norms, and moral norms, we found that attitude (OR 9.16, 95% CI 4.02-20.85), moral norm (OR 7.97, 95% CI 4.49-14.14), descriptive norm (OR 2.83, 95% CI 1.63-4.92), and anticipated regret (OR 2.43, 95% CI 1.71-3.46) were significant factors of pregnant women’s intention (Table 3). Comparison of deviance showed that the model that included additional sociocognitive variables better explained pregnant women’s intention (∆ deviance=41.33, *P*>.05, Table 3).

To investigate whether health literacy predicted pregnant women’s intention in our theoretical model, we sequentially added each health literacy variable to the ordinal logistic regression model and observed that the pregnant women’s intention was not affected by health literacy (Table 3). In addition, no interaction term was identified between health literacy variables and direct constructs determining intention. We performed structural equation modeling in parallel with our stepwise regression model and observed the same results (data not shown but available from authors).

To identify significant underlying beliefs, we performed another ordinal logistic regression model with beliefs related to attitude, as attitude was the only significant construct with underlying beliefs. We found 3 significant beliefs related specifically to the attitude construct, namely, that the use of a DA (1) would reassure pregnant women (OR 2.55, 95% CI 1.73-4.01), (2) would facilitate their reflection with their spouse (OR 1.55, 95% CI 1.05-2.29), and (3) would let them know about the advantages of doing or not doing a prenatal screening test for Down syndrome (OR 1.53, 95% CI 1.05-2.24; Table 4).

**Table 3:** Significant determinants of pregnant women’s intention (n=346).

Construct	Odds ratio (95% CI)					
	TPB^a^	Extended TPB	Extended TPB and subjective numeracy	Extended TPB and subjective literacy	Extended TPB and objective numeracy	Extended TPB and objective literacy^b^
Attitude	13.38 (6.40-27.90)	9.16 (4.02-20.85)	9.13 (4.00-20.84)	9.26 (4.06-21.11)	9.58 (4.14-22.12)	N/A^c^
Subjective norm	3.64 (2.33-5.70)	0.91 (0.51-1.61)	0.91 (0.51-1.61)	0.90 (0.50-1.60)	0.89 (0.50-1.59)	N/A
Perceived control	2.36 (1.43-3.90)	1.69 (0.92-3.08)	1.68 (0.91-3.09)	1.75 (0.95-3.22)	1.65 (0.89-3.03)	N/A
Anticipated regret	N/A	2.43 (1.71-3.46)	2.44 (1.70-3.48)	2.33 (1.61-3.36)	2.47 (1.73-3.52)	N/A
Descriptive norm	N/A	2.83 (1.63-4.92)	2.83 (1.63-4.92)	2.82 (1.62-4.90)	2.84 (1.64-4.93)	N/A
Moral norm	N/A	7.97 (4.49-14.14)	7.97 (4.49-14.15)	8.38 (4.65-15.06)	7.92 (4.46-14.08)	N/A
Health literacy^d^	N/A	N/A	1.02 (0.58-1.81)	0.78 (0.43-1.43)	1.18 (0.66-2.12)	N/A
Deviance	316.78	358.11	358.10	357.48	357.80	N/A
∆ deviance		41.33	0.01	0.63	0.31	N/A
*P* value		<.001	.92	.43	.58	N/A

^a^TPB: Theory of Planned Behavior.

^b^Objective literacy could not be added to the regression model because of the lack of variability in the distribution.

^c^N/A: not applicable.

^d^Subjective numeracy: score≥ median versus score < median; subjective health literacy: adequate versus inadequate; objective numeracy: all correct answers versus one error or more.

**Table 4:** Significant beliefs of pregnant women (n=346).

Construct	Underlying belief	Descriptive analysis		Odds ratio (95% CI)
		Mean^a^ (SD)	Median^a^ (Q1-Q3)	
Attitude	Emotions: the use of a DA^b^ would reassure pregnant women	3.85 (0.96)	4.00 (3.00-5.00)	2.55 (1.73-4.01)
	Advantages: the use of a DA would facilitate their reflection with their spouse	4.15 (0.91)	4.00 (4.00-5.00)	1.55 (1.05-2.29)
	Advantages: the use of a DA would let them know about the advantages of doing or not doing the prenatal screening test for DS^c^	4.28 (0.94)	4.00 (4.00-5.00)	1.53 (1.05-2.24)
Anticipated regret	N/A^d^	2.95 (1.04)	3.00 (2.00-4.00)	2.06 (1.47-2.88)
Descriptive norm	N/A	3.79 (0.80)	4.00 (3.33-4.33)	2.73 (1.62-4.58)
Moral norm	N/A	4.05 (0.87)	4.00 (3.67-5.00)	8.86 (5.19-15.14)

^a^Out of 5.

^b^DA: decision aid.

^c^DS: Down syndrome.

^d^N/A: not applicable.

## Discussion

### Principal Findings

In this theory-based Web survey, we sought to identify psychosocial factors influencing pregnant women’s intention to use a DA for prenatal screening for Down syndrome and assessed whether health literacy added to the predictive power of this model. There are no data specifying the profile of pregnant women in the province of Quebec, but our sample compared well to that of women in the province overall, except for education and health literacy levels, which were higher in our sample [85-87]. Overall, we found that pregnant women showed high levels of intention to use a DA for prenatal screening for Down syndrome. Also, we observed that, in order of importance, attitude, moral and descriptive norms, and anticipated regret were the factors that explained most of their behavioral intention. In other words, the perception of the advantages of using a DA (attitude), the possible regret foreseen if the DA is not used (anticipated regret), the perception that it is a common practice (descriptive norm), and the feeling that using a DA for this decision would be in agreement with their moral values (moral norm) were significantly associated with a strong intention to use the DA. In addition, we identified 3 attitudinal beliefs significantly associated with women’s intention: perceiving that using a DA (1) would reassure them, (2) would facilitate their reflection with their spouse, and (3) would let them know about the advantages of doing or not doing a prenatal screening test for Down syndrome. On the other hand, our findings showed that neither health literacy levels nor individual sociodemographic characteristics had any influence on the behavioral intention of interest, suggesting that, regardless of their health literacy levels and sociodemographic characteristics, all women are under the influence of the same sociocognitive factors regarding whether or not they intend to use a DA for prenatal screening for Down syndrome. These findings lead us to make 5 main points with regards to pregnant women’s intention, the direct determinants of their intention, their underlying beliefs, the influence of health literacy, and the next steps.

### Comparison With Prior Work

First, to the best of our knowledge, this study is among the first to adopt an all-encompassing theory-based approach to identifying factors, including health literacy, influencing the intention to use a DA in prenatal care. Our results support earlier research on SDM implementation indicating that women showed a strong intention to engage in SDM regarding prenatal screening for Down syndrome [26]. Moreover, this high level of intention may reflect a need felt by pregnant women facing prenatal screening choices to become more skilled in discussing screening tests with their health care provider, which is congruent with the literature on pregnant women’s decision-making needs [4,6,9]. This strong intention suggests that future efforts to increase DA use and SDM among clinicians for prenatal screening for Down syndrome would find a favorable response in pregnant women.

Second, we observed that the following factors, in order of importance, influenced pregnant women’s intention: attitude, moral norm, descriptive norm, and anticipated regret. These findings are congruent with earlier research on SDM implementation in this context [26], which showed that attitude, subjective norm, self-efficacy, and moral norm were determinants of pregnant women’s intention to engage in SDM. Although the variables “descriptive norm” and “anticipated regret” were not investigated in the earlier study, the influence of social pressure came out through the subjective norm variable, which refers to the influence of significant individuals in women’s entourage. Contrary to the findings of our study, and despite the similarity of the samples, however, the previous study showed self-efficacy as a determining factor among women without postsecondary education (although not among women with a higher level of education), whereas in our study sample perceived control was not a significant factor. Self-efficacy and perceived control are not the same constructs, but they are closely related as they both refer to a person’s evaluation of the degree of difficulty of adopting a given behavior. This difference in findings could reflect the fact that the earlier study considered intention to *engage in SDM* while our study asked women about their intention to *use a DA*, a practice that constitutes one specific behavior in the overall SDM process. Pregnant women with low education could have more confidence (self-efficacy) about using a DA than about engaging in SDM in general, belying the common myth that the use of DAs is equivalent to the behavior of engaging in SDM [12,88].

Third, significant salient beliefs underlying attitude were, in order of importance, (1) the women’s belief that the use of a DA would reassure them; (2) the belief that it would facilitate their reflection with their spouse; and (3) the belief that it would let them know about the advantages of doing or not doing the prenatal screening test for Down syndrome. These results are congruent with earlier research on decisional needs among pregnant women facing prenatal screening for Down syndrome, which showed that the main difficulties perceived by pregnant women were pressure from others, emotions, and lack of information [4]. Our findings provide information about modifiable attitudinal beliefs regarding DA use that could facilitate design of implementation strategies to increase their use by pregnant women in clinical practice. According to the Intervention Mapping approach, efficient interventions should “contain specific messages that target selected beliefs within the determinants of interest, and require specific translation to practical applications to reach optimal fit” [23]. In practice, a public health communication campaign combined with interventions mediated by health professionals could reinforce the influence of women’s attitude to DA use by targeting its 3 identified underlying beliefs (reassurance, reflection with spouse, awareness of advantages of each choice). Key statements regarding these 3 salient beliefs could also be added to the DA to increase women’s intention to use it and help clinicians to explain it.

Fourth, health literacy was not a factor that influenced women’s intention in our study, although many studies have shown associations between health literacy and related notions, such as patient involvement in decision making [27,38,40-43,47,68]. Hence, a single population based DA implementation program would benefit any pregnant women in the province of Quebec to help them make informed values-congruent decision about prenatal screening for Down syndrome. To the best of our knowledge, this study is among the first to investigate the role of health literacy within the context of a theory-driven study using a behavior change model. Our findings suggest that regardless of their perceived or actual capacity to understand complex information, pregnant women would like to use a DA to decide about prenatal screening for Down syndrome. In addition, in our bivariate analyses we did identify some interesting associations between health literacy and perceived control. Although perceived control was not a predictive factor in our study, this association suggests that in contexts where it does play a predictive role, such as intention to engage in healthy eating behaviors [89], intention may be affected by health literacy. Similar studies that include populations with low health literacy should be conducted before decisive claims can be made. However, some have argued that it would be more efficient to work at clearer health communication and fostering participation among all patients rather than screening them for health literacy [90]. Our results should not be used to minimize the importance of improving patients’ understanding. It is of primary importance to communicate clearly with every pregnant woman about Down syndrome screening and invite them to ask questions, whatever their health literacy level.

Finally, if the findings of this study are valid, identifying the 4 most significant determinants of pregnant women’s intention to use a DA to decide about Down syndrome screening will be useful for the design of interventions to promote uptake. Each of the 4 determinants can be mapped to the Behavior Change Wheel [91], a method developed to inform the design of behavior change interventions. This generates a set of recommended interventions (such as education, training programs, persuasion, modelling, etc), each with its appropriate related methods. For example, clinicians could be trained to introduce the advantages of using the DA, and present the use of DAs as a common practice. Indeed, while it has been shown that lack of training was an important barrier to DA implementation [17], pregnant women indicated that clinicians had a key role in delivering such information [92]. An implementation intervention could also consist of a Web-based application coupled with a DA, which would enrich the current bank of online decision support tools [93]. In terms of moral norm, our findings confirm that there is a significant ethical dimension to the decision about prenatal screening [94-96]. No psychological techniques have yet been formally identified for considering the influence of the moral norm in an intervention [97]. Also, it has been shown that interventions that aim to manipulate moral norms can be counterproductive because of a “boomerang effect” that arises when a person perceives that his or her freedom is threatened by social pressure [98,99]. Health professionals could thus simply be invited to explain to women why moral values are at stake in the decision, so that they can subsequently discuss this and clarify the decision in light of the woman’s moral principles.

### Limitations

This study has limitations. First, although we suspected that the lack of variability in our health literacy findings could have been due to lack of scale discriminating capacity in our sample, except for the objective health literacy scale (S-TOFHLA), the administered scales covered many dimensions of health literacy and correlated together, indicating a convergence of the results (see Multimedia Appendix 7). In addition, this study will enable further validation of new French versions of the 3 scales.

Second, it is possible that the video used to present the behavior of using a DA mediated pregnant women’s intent. However, we felt it was more important to ensure that respondents understood the nature of the behavior being studied than to avoid any risk of mediated intent by not using a video at all.

Third, use of the TPB framework could in itself have shaped our findings. For example, the assumption that agents and their actions are rational may neglect the role of nonrational factors (such as emotion or experience) in human action and reasoning. Also, the approach underestimates the singularity of each agent, as it considers that similar agents are influenced by similar factors [100]. Despite these theoretical limitations, from the wider perspective of developing potentially effective implementation strategies, a TPB-based approach promotes the collection of comprehensive, consistent and valid information, and is still one of the most frequently applied theories in the domain of behavior change.

Finally, we cannot assume that the results can be extrapolated widely without further research. Pregnant women in the sample were mostly white, with French as their mother tongue. This may weaken the external validity of our sample, but not with regards to the general population of the province of Quebec, which was the aim of this study. Because Canadian health care services are organized into 10 provincial and 3 territorial health care systems, each requires its own population-based prenatal screening program. Also, pregnant women in our sample were highly educated compared with women in the province of Quebec overall, where less than a quarter of women aged from 15 to 44 years old have a university education [87]. Likewise, health literacy levels were very high overall, far above that of the general population of the province of Quebec but also of the rest of Canada, where more than half the population has inadequate levels [85,86]. Pregnant women in our sample were recruited from a large Web panel and, as participants willingly subscribed to the panel, their literacy levels, including health literacy and eHealth literacy, might be higher than that of the general population [101]. Our study sample may thus lack representativeness with regard to vulnerable and less literate populations.

Our recruitment methods reflected our main objective (psychosocial determinants of women’s intention), but methods in future studies looking at health literacy should be informed by the specificities of the studied population with respect to education levels, Internet use and eHealth literacy.

### Conclusions

This study, based on a theoretical approach to behavior change, indicated which factors will need to be addressed to design an effective implementation intervention for the use of DAs in the context of prenatal screening for Down syndrome. Our findings indicate that women’s intention to use a DA in this context was determined by the consequent pros and cons they perceived (attitude), its compatibility with their moral values (moral norm), their perception of how much other women use it (descriptive norm), and the regret they perceive they might feel if they do not use it (anticipated regret). This study provides valuable and specific guidance for designing an intervention to implement the use of a DA and ultimately to foster SDM in prenatal care.

## Appendix

Multimedia Appendix 1 Online questionnaire.

Multimedia Appendix 2 Decision aid for prenatal screening for Down syndrome.

Multimedia Appendix 3 Socio-cognitive variables scores and correlations.

Multimedia Appendix 4 Intention level by sociodemographic characteristics.

Multimedia Appendix 5 Bivariate analysis of health literacy by intention category.

Multimedia Appendix 6 Associations between health literacy scores and socio-cognitive factors.

Multimedia Appendix 7 Health literacy measure correlations.

## Abbreviations

CHERRIES: Checklist for Reporting Results of Internet E-Surveys
DA: decision aid
NIPT: noninvasive prenatal test
OR: odds ratio
PEGASUS: Personalized Genomics for Prenatal Aneuploidy Screening Using Maternal Blood
SDM: shared decision making
SNS: Subjective Numeracy Scale
S-TOFHLA: Short Test of Functional Health Literacy in Adults
3HLQ: 3 health literacy questions
3NQ: 3 numeracy questions
TPB: Theory of Planned Behavior
