# Supplementary material for: Role of Psychosocial Factors and Health Literacy in Pregnant Women’s Intention to Use a Decision Aid for Down Syndrome Screening: A Theory-Based Web Survey
Source: J Med Internet Res. 2016 Oct 28;18(10):e283. doi: 10.2196/jmir.6362 (PMC5106559; doi:10.2196/jmir.6362)
Supplement: Multimedia Appendix 1 [file jmir_v18i10e283_app1.pdf]

Select Question(Link)

SurveyInfo

ONLY DISPLAYED IN TEST MODE

STATUS:
StatusLeger: incomplet
INTCODE: Incomplete

VERSION:
VERSI: 1

LINK INFO:
IDCON: p4379613
SLINK: https://ci.legerweb.com//extwix/test_p4379613.aspx?__etk=VASGCTRRPIYU&r=262&s=HAPSOCDP
SID: HAPSOCDP
QID: 0000000262

REDIRECTS:
ECHAN: *NO RESPONSE*
REDIR: https://Legerweb.com/ES.asp?Imid=&CodeSurvey=&l=&IdCamp=&Flag=C
REDI: https://Legerweb.com/ES.asp?Imid=&CodeSurvey=&l=&IdCamp=&Flag=S
REDIQ: https://Legerweb.com/ES.asp?Imid=&CodeSurvey=&l=&IdCamp=&Flag=Q
REDIRTXT: Please click on the following arrow to receive your reward(s).

RESPONDENT DEVICE INFO:
BrowserName:
BrowserType: Chrome
BrowserVersion: 45.0
IP Address: 132.203.245.9
User Agent: Mozilla/5.0 (Windows NT 6.1; WOW64) AppleWebKit/537.36 (KHTML, like Gecko) Chrome/45.0.2454.85 Safari/537.36
Device Type: Laptop/PC
Survey Rendering Mode: Desktop: Desktop rendering (PC/Mac/tablet)
Operating System: Windows 7

SURVEY LAYOUT:
CurrentSurveyLayout: Main theme - with Title

>>

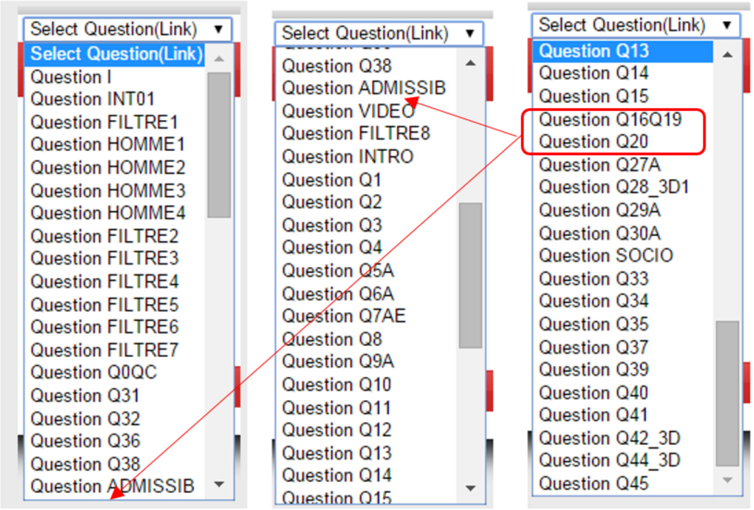

Select Question(Link) ▼

Survey language

Préférez-vous répondre à ce questionnaire en anglais ou en français ?

Would you prefer to complete the survey in English or French?

◯ English (Anglais)
◯ Français (French)

>>

Select Question(Link) ▼

INT01 - ASK ALL

This province-wide survey is directed by a research team in public health at Université Laval, Quebec city, Quebec. Your answers are important for helping us improve a sector of your health care. Therefore, do you agree to answer the following survey questions truthfully and thoughtfully?

◯ I agree
◯ I disagree

>>

Select Question(Link) ▼

FILTRE1

Are you...

◯ A man
◯ A woman

>>

Filters for men:

Select Question(Link) ▼

HOMME1

Do you have a female partner?

◯ Yes
◯ No

>>

Select Question(Link) ▼

HOMME2

How old is she?

◯ Less than 18 years old
◯ 18 to 44 years old
◯ 45 to 70 years old
◯ More than 70 years old

>>

Select Question(Link) ▼

HOMME3

Is she...

Please check everything that applies to her.

- ☐ Pregnant
- ☐ Diabetic
- ☐ Menopausal
- ☐ Asthmatic
- ◯ None of the above

>>

Select Question(Link) ▼

HOMME4

Your partner may be eligible to do this survey, which takes approximately 30 minutes to complete. If she is eligible to answer the whole questionnaire, she will get $25.

If your partner is not available right now, you can close the window, and come back later with her by clicking on the same link.

Thank you for your collaboration.

Please click on the following arrow if your partner wants to continue this survey on her own.

- ☐ My partner doesn't want to participate

>>

Select Question(Link) ▼

FILTRE2

How old are you?

- ◯ Less than 18 years old
- ◯ 18 to 44 years old
- ◯ 45 to 70 years old
- ◯ More than 70 years old

>>

Filters for women:

Select Question(Link) ▼

FILTRE2

How old are you?

- ◯ Less than 18 years old
- ◯ 18 to 44 years old
- ◯ 45 to 70 years old
- ◯ More than 70 years old

>>

Select Question(Link) ▼

FILTRE3

Are you...

Please check everything that applies to you.

- ☐ Pregnant
- ☐ Diabetic
- ☐ Menopausal
- ☐ Asthmatic
- ◯ None of the above

>>

Select Question(Link) ▼

FILTRE4

How many weeks have you been pregnant?

- ◯ Less than 16 weeks
- ◯ 16 to 30 weeks
- ◯ More than 30 weeks

>>

Select Question(Link) ▼

FILTRE5

Have you already made a decision about doing a prenatal screening test for trisomy 21 or not?

- ◯ I have decided to do a prenatal screening test for trisomy 21.
- ◯ I have decided NOT to do a prenatal screening test for trisomy 21.
- ◯ I have made my decision but I would rather not reveal my choice.
- ◯ I still not have decided if I will do a prenatal screening test for trisomy 21 or not.

>>

Select Question(Link) ▼

FILTRE6

Is your pregnancy is considered a « high-risk pregnancy »? In other words, do you have gestational diabetes, hypertension, risk of miscarriage or premature delivery, eclampsia or a multiple pregnancy (ex: twins, triplets)?

- ◯ I have a high-risk pregnancy
- ◯ I don't have a high-risk pregnancy

>>

Select Question(Link) ▼

FILTRE7

During a previous pregnancy or during your current pregnancy, did you participate in a research project in which we asked you to view this video (see the three (3) representative images below)?

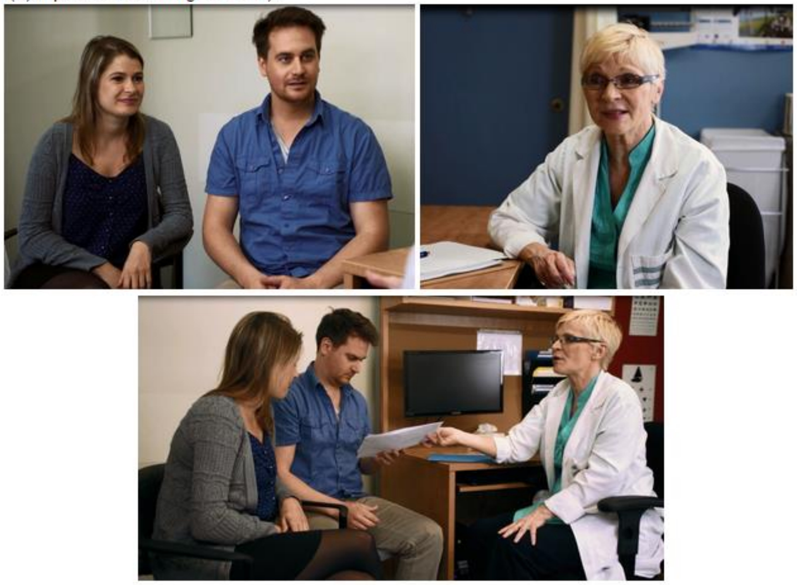

- ◯ I already saw this video
- ◯ I never saw this video
- ◯ I don't know

>>

Select Question(Link)

Q0QC - ASK ALL

In which region of Quebec do you live?

- ◯ Bas-Saint-Laurent
- ◯ Saguenay-Lac-Saint-Jean
- ◯ Capitale-Nationale
- ◯ Mauricie
- ◯ Estrie
- ◯ Montréal
- ◯ Outaouais
- ◯ Abitibi-Témiscamingue
- ◯ Côte-Nord
- ◯ Nord-du-Québec
- ◯ Gaspésie/Îles-de-la-Madeleine
- ◯ Chaudière-Appalaches
- ◯ Laval
- ◯ Lanaudière
- ◯ Laurentides
- ◯ Montérégie
- ◯ Centre-du-Québec
- ◯ Other, please specify: ____________
- ◯ I don't know

>>

Select Question(Link)

Q31

What is your date of birth?

Day	Month	year
Please select your answer	Please select your answer	Please select your answer

>>

Select Question(Link)

Q32

What is your ethnic background?

- ◯ White/Caucasian
- ◯ Black/African/Afro-American, etc.
- ◯ Native/Autochthon/First nations
- ◯ Latin American (Mexico, Chile, Costa Rica, etc.)
- ◯ Arab (Middle East, Maghreb)
- ◯ South Asian (India, Bangladesh, Pakistan, Sri-Lanka, etc.)
- ◯ Southeast Asian (Vietnam, Cambodia, Malaysia, Laos, etc.)
- ◯ West Asian (Iran, Afghanistan, etc.)
- ◯ Chinese
- ◯ Filipino
- ◯ Korean
- ◯ Japanese
- ◯ Other, please specify: ____________
- ◯ I would rather not answer

>>

Select Question(Link) ▼

Q36

What is the highest level of education for which you OBTAINED a diploma?

- ◯ No secondary education
- ◯ Secondary education
- ◯ Vocational studies (DVS, ACS, etc.)
- ◯ Collegial studies
- ◯ University studies
- ◯ Other, please specify: ____________

>>

Select Question(Link) ▼

Q38

What is your approximate annual household income?

- ◯ Less than $29,999
- ◯ $30,000 to $59,999
- ◯ $60,000 to $99,999
- ◯ $100,000 or more
- ◯ I don't know
- ◯ I would rather not answer

>>

Select Question(Link) ▼

ADMISSIB

You are eligible to participate in this study available only for pregnant women.

First, please answer to some questions about numerical information and a few health-related questions.

Next, you will be invited to watch a video of 10 minutes. You must watch the whole video and then complete the questionnaire (about 20 minutes).

To get the $25, you must complete the whole questionnaire.

At any moment you can interrupt the session and come back later by clicking on the same link.

Please click on the following arrow to start the questionnaire.

>>

Select Question(Link)

Q16Q19

For the next four (4) questions, please check the box that best reflects how good you are at doing the following things:

	Not at all good				Extremely good
How good are you at working with fractions?	◯	◯	◯	◯	◯
How good are you at working with percentages?	◯	◯	◯	◯	◯
How good are you at calculating a 15% tip?	◯	◯	◯	◯	◯
How good are you at figuring out how much a shirt will cost if it is 25% off?	◯	◯	◯	◯	◯

>>

Select Question(Link)

Q20

For the next seven (7) questions, please check the box that **best reflects your answer**:

	Not at all helpful				Extremely helpful
When reading the newspaper, how helpful do you find tables and graphs that are parts of a story?	◯	◯	◯	◯	◯

	Always prefer words				Always prefer numbers
When people tell you the chance of something happening, do you prefer that they use words ("it rarely happens") or numbers ("there's a 1% chance")?	◯	◯	◯	◯	◯

	Always prefer percentages				Always prefer words
When you hear a weather forecast, do you prefer predictions using percentages (e.g., "there will be a 20% chance of rain today") or predictions using only words (e.g., "there is a small chance of rain today")?	◯	◯	◯	◯	◯

	Never				Very often
How often do you find numerical information to be useful?	◯	◯	◯	◯	◯

How confident are you filling out forms by yourself?

Never confident	Rarely confident	Sometimes confident	Often confident	Always confident
○	○	○	○	○

How often do you have someone help you read hospital materials?

Always	Often	Sometimes	Rarely	Never
○	○	○	○	○

How often do you have problems learning about your medical condition because of difficulty reading hospital materials?

Always	Often	Sometimes	Rarely	Never
○	○	○	○	○

>>

Select Question(Link) ▼

VIDEO

Here is a video showing the best way to use a **"Decision aid (written document of 4 pages)"** by a couple and their family physician when considering doing or not prenatal screening for Down syndrome (or trisomy 21). In the following survey, we want to find out **your opinion** on the use of this tool **if you ever had** to use it in the future.

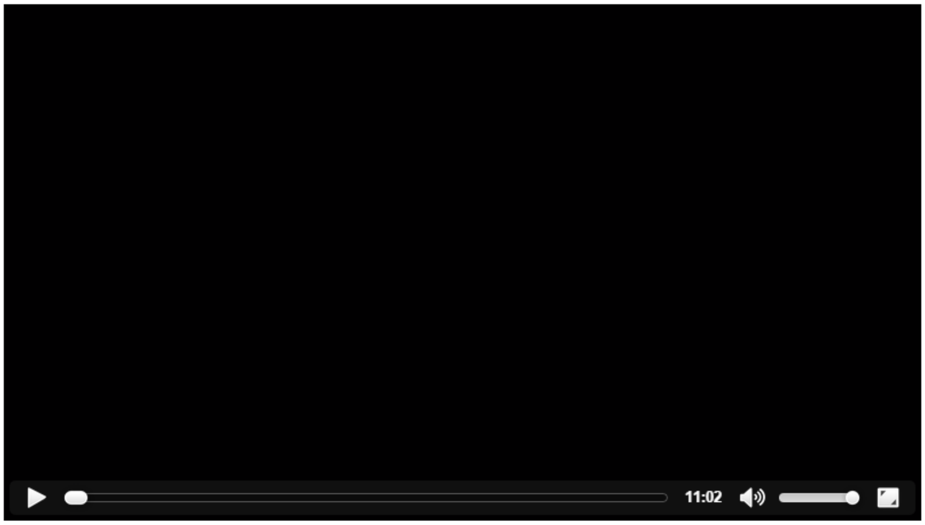

After watching the whole video, an arrow will appear below and you will be able to start the questionnaire.

Select Question(Link)

FILTRE8 - ASK IF FILTRE7=I don't know

Because you were unsure, can you confirm if you have ever seen this video before or not?

- ◯ I have seen this video before
- ◯ I have never seen this video before

>>

Select Question(Link)

INTRO

Health professionals who monitor pregnancies must inform pregnant women about tests available to screen for Down syndrome (or trisomy 21). Most people want to be involved in decision making about their health. The use of a decision aid can increase the active involvement of pregnant women in making an informed and shared decision that respects their values and preferences.

The video that you just watched showed the best way to use a **"Decision aid (written document of 4 pages)"** by a couple and their family physician when considering prenatal screening for Down syndrome. The goal of the following questionnaire is **to find out your opinion on the use of this decision aid if you ever had to use it in the future**.

In parallel, we are doing a similar project with health professionals who monitor pregnancies to find out their opinion. The objective is to introduce routine use of a decision aid that is adequate and useful for everyone.

Please note:

1. For each question, please **check the box** which best corresponds to your answer. There are no right or wrong answers. It is **your opinion** that interests us.
2. Certain statements may seem repetitive. This is due to the study's methodology. Please answer **all** questions.
3. It is important that you complete the whole questionnaire **alone**, without any help.
4. By completing this survey, you give your **consent** to participate in this research project.
5. At the end of the questionnaire, we will ask you to answer a few health-related questions and a few sociodemographic questions.

Your participation is much appreciated, thank you.

Team of the Canada Research Chair in Implementation of Shared Decision Making in Primary Care. Dr. France Légaré, MD, PhD, clinician researcher and titular professor, CHUQ Research Centre, Department of Family Medicine and Emergency Medicine, Faculty of Medicine, Université Laval, Quebec City, Quebec, Canada.

This project is funded by a research grant from Genome Canada and Genome Quebec and was approved by the ethical review boards of CHU de Québec, CSSS Vieille-Capitale and CSSS Alphonse-Desjardins.

Please click on the following arrow to start the questionnaire.

>>

Select Question(Link) ▼

Q1

In the context of prenatal screening for trisomy 21, using a decision aid...

	Very unlikely	Somewhat unlikely	Neither unlikely or likely	Somewhat likely	Very likely
...would help me learn the advantages of doing or not the screening test.	◯	◯	◯	◯	◯
...would help me make an informed decision.	◯	◯	◯	◯	◯
...would provide me with too much information.	◯	◯	◯	◯	◯
...would provide me with useful information.	◯	◯	◯	◯	◯
...would help me learn the disadvantages of doing or not the screening test.	◯	◯	◯	◯	◯
...would facilitate reflection with my spouse.	◯	◯	◯	◯	◯

>>

Select Question(Link) ▼

Q2

In the context of prenatal screening for trisomy 21, it would be easier for me to use a decision aid...

	Strongly disagree	Somewhat disagree	Neither disagree or agree	Somewhat agree	Strongly agree
...if it was given to me by my healthcare professional monitoring my pregnancy (example: physician, midwife, nurse, etc.).	◯	◯	◯	◯	◯
...if it was available online.	◯	◯	◯	◯	◯
...if it was available in print.	◯	◯	◯	◯	◯

>>

Select Question(Link) ▼

Q3

In the context of prenatal screening for trisomy 21, the following people would approve/disapprove of my using a decision aid:

	Would strongly disapprove	Would somewhat disapprove	Would neither disapprove or approve	Would somewhat approve	Would strongly approve
my spouse	◯	◯	◯	◯	◯
the healthcare professional monitoring my pregnancy (example: physician, midwife, nurse, etc.)	◯	◯	◯	◯	◯
my parents	◯	◯	◯	◯	◯
my family (other than my parents and my spouse)	◯	◯	◯	◯	◯
my friends	◯	◯	◯	◯	◯

>>

Select Question(Link) ▼

In the context of prenatal screening for trisomy 21...

in your view, what percentage of pregnant women around you would use a decision aid.

None 0%	A quarter 25%	Half 50%	Three quarters 75%	All 100%
◯	◯	◯	◯	◯

>>

Femmes #1 à 44 :

Select Question(Link) ▼

Q5A

In the context of prenatal screening for trisomy 21...

	Very unlikely	Somewhat unlikely	Neither unlikely or likely	Somewhat likely	Very likely
...I would regret NOT having used a decision aid	◯	◯	◯	◯	◯

	Strongly disagree	Somewhat disagree	Neither disagree or or agree	Somewhat agree	Strongly agree
...the need to be informed would motivate me to use a decision aid.	◯	◯	◯	◯	◯
...I would be more motivated to use a decision aid if my healthcare professional showed interest in presenting it to me.	◯	◯	◯	◯	◯
...the need to make an informed decision would lead me to use a decision aid.	◯	◯	◯	◯	◯
...I believe that most of the pregnant women around me would use a decision aid.	◯	◯	◯	◯	◯

	Very unlikely	Somewhat unlikely	Neither unlikely or likely	Somewhat likely	Very likely
...I would be fine NOT using a decision aid.	◯	◯	◯	◯	◯

>>

Femmes #45 et plus

Select Question(Link) ▼

Q5A

In the context of prenatal screening for trisomy 21...

	Very unlikely	Somewhat unlikely	Neither unlikely or likely	Somewhat likely	Very likely
...I would regret NOT having used a decision aid	◯	◯	◯	◯	◯

	Strongly disagree	Somewhat disagree	Neither disagree or or agree	Somewhat agree	Strongly agree
...the need to be informed would motivate me to use a decision aid.	◯	◯	◯	◯	◯
...I would be more motivated to use a decision aid if my healthcare professional showed interest in presenting it to me.	◯	◯	◯	◯	◯
...the need to make an informed decision would lead me to use a decision aid.	◯	◯	◯	◯	◯
...I believe that most of the pregnant women around me would use a decision aid.	◯	◯	◯	◯	◯

	Very unlikely	Somewhat unlikely	Neither unlikely or likely	Somewhat likely	Very likely
...I would be fine NOT using a decision aid.	◯	◯	◯	◯	◯
...if I wanted to, I could easily use a decision aid.	◯	◯	◯	◯	◯

>>

Select Question(Link) ▼

Q6A

In the context of prenatal screening for trisomy 21...

	Very difficult	Somewhat difficult	Neither difficult or easy	Somewhat easy	Very easy
...using a decision aid would be:	◯	◯	◯	◯	◯

	Very detrimental	Somewhat detrimental	Neither detrimental or beneficial	Somewhat beneficial	Very beneficial
...using a decision aid would be:	◯	◯	◯	◯	◯

	Very dull	Somewhat dull	Neither dull or interesting	Somewhat interesting	Very interesting
...using a decision aid would be:	◯	◯	◯	◯	◯

	Very unwise	Somewhat unwise	Neither unwise or wise	Somewhat wise	Very wise
...using a decision aid would be:	◯	◯	◯	◯	◯

	Very unenjoyable	Somewhat unenjoyable	Neither unenjoyable or enjoyable	Somewhat enjoyable	Very enjoyable
...using a decision aid would be:	◯	◯	◯	◯	◯

	Very useless	Somewhat useless	Neither useless or useful	Somewhat useful	Very useful
...using a decision aid would be:	◯	◯	◯	◯	◯

	Very unpleasant	Somewhat unpleasant	Neither unpleasant or pleasant	Somewhat pleasant	Very pleasant
...using a decision aid would be:	◯	◯	◯	◯	◯

>>

Select Question(Link) ▼

Q7AE

In the context of prenatal screening for trisomy 21...

	Stronly disagree	Somewhat disagree	Neither disagree or agree	Somewhat agree	Strongly agree
...most people who are important to me would recommend that I use a decision aid.	○	○	○	○	○
...I would feel that I am able to use a decision aid.	○	○	○	○	○
...the people who are most important to me would think that I should use a decision aid.	○	○	○	○	○
...I believe that many pregnant women around me would use a decision aid.	○	○	○	○	○
..it would be up to me to use a decision aid.	○	○	○	○	○

	Would strongly disapprove	Would somewhat disapprove	Would neither disapprove or approve	Would somewhat approve	Would strongly approve
...if I used a decision aid, most of the people who are important to me:	○	○	○	○	○

>>

Select Question(Link) ▼

Q8

As a pregnant woman, in the context of prenatal screening for trisomy 21...

	Stronly disagree	Somewhat disagree	Neither disagree or agree	Somewhat agree	Strongly agree
...I consider that it would be appropriate for me to use a decision aid.	○	○	○	○	○
...I consider that it would be my responsibility to use a decision aid.	○	○	○	○	○
...I consider that it would be necessary for me to use a decision aid.	○	○	○	○	○

>>

Select Question(Link) ▼

Q9A

In the context of prenatal screening for trisomy 21...

	Very unlikely	Somewhat unlikely	Neither unlikely or likely	Somewhat likely	Very likely
...I would use a decision aid.	○	○	○	○	○

	Strongly disagree	Somewhat disagree	Neither disagree or agree	Somewhat agree	Strongly agree
...using a decision aid would be in keeping with my moral values.	○	○	○	○	○

	Very unlikely	Somewhat unlikely	Neither unlikely or likely	Somewhat likely	Very likely
...even if it was difficult, I would be able to use a decision aid.	○	○	○	○	○

	Strongly disagree	Somewhat disagree	Neither disagree or agree	Somewhat agree	Strongly agree
...using a decision aid would be in keeping with my principles.	○	○	○	○	○

	Very unlikely	Somewhat unlikely	Neither unlikely or likely	Somewhat likely	Very likely
...I would intend on using a decision aid.	○	○	○	○	○

	Strongly disagree	Somewhat disagree	Neither disagree or agree	Somewhat agree	Strongly agree
...my personal values would lead me to use a decision aid.	○	○	○	○	○

	Very low	Quite low	Neither low or strong	Quite strong	Very strong
...the likelihood of my using a decision aid is:	○	○	○	○	○

>>

Select Question(Link) ▼

Q10

In the context of prenatal screening for trisomy 21, I would feel capable of using a decision aid...

	Very unlikely	Somewhat unlikely	Neither unlikely or likely	Somewhat likely	Very likely
...even if my healthcare professional did not have enough time to present it to me.	○	○	○	○	○
...even if it is difficult to understand.	○	○	○	○	○
...even if it did NOT include information about the prenatal tests offered by the private sector.	○	○	○	○	○
...even if my decision regarding the screening test was already made.	○	○	○	○	○

>>

Select Question(Link)

Q11

In the context of prenatal screening for trisomy 21, using a decision aid...

	Very unlikely	Somewhat unlikely	Neither unlikely or likely	Somewhat likely	Very likely
...would reassure me.	◯	◯	◯	◯	◯
...would make me anxious.	◯	◯	◯	◯	◯

>>

Select Question(Link)

Q12

In the context of prenatal screening for trisomy 21, I already knew a decision aid:

◯ Yes
◯ No

>>

Select Question(Link)

Q13

For the next three (3) questions, please answer the best you can, without using a calculator and without any help:

A person who takes medication "A" has 1% chance of having an allergic reaction. If 1000 people take medication "A", how many will have an allergic reaction?

Your answer: ______ person(s) out of 1000

A person who takes medication "B" has 1 chance in 1000 of having an allergic reaction. What percentage of people who take medication "B" will have an allergic reaction?

Your answer: ______ %

Imagine that you toss a coin 1000 times for heads or tails. At your best guess, how many times out of 1000 will you get heads?

Your answer: ______ times out of 1000

>>

Select Question(Link) ▾

Below are some common medical and administrative instructions. For the next sections, please choose the word which suits best for the sentences to make sense.

Preparation for the radiography (X-ray)

Your doctor has sent you to have a [▾] X-ray.

You must have an [▾] stomach when you come for [▾].

The X-ray will [▾] from 1 to 3 [▾] to do.

>>

Select Question(Link) ▾

Q28_3D1

Below are some common medical and administrative instructions. For the next sections, please choose the word which suits best for the sentences to make sense.

The day before the radiography (X-ray)

For supper have only a [▾] snack of fruit, [▾] and jelly, with coffee or tea.

After [▾], you must not [▾] or drink anything at [▾] until after you have [▾] the X-ray.

>>

Select Question(Link) ▾

Below are some common medical and administrative instructions. For the next sections, please choose the word which suits best for the sentences to make sense.

The day of the radiography (X-ray)

Do not eat [▾].

Do not [▾], even [▾].

If you have any [▾], call the X-ray [▾].

>>

Select Question(Link) ▼

Below are some common medical and administrative instructions. For the next sections, please choose the word which suits best for the sentences to make sense.

Eligibility for the Québec Health Insurance Plan or QHIP (In French: RAMQ)

I agree to give correct information to [▼] if I am eligible for the Québec Health Insurance Plan (or QHIP).

I [▼] to provide to the QHIP information to [▼] any statements given in

this [▼] and hereby give permission to the [▼] to get such proof.

I [▼] that for the QHIP I must report any [▼] in my circumstances within

[▼] (10) days of becoming [▼] of the change.

I understand [▼] if I DO NOT like the [▼] made on my case, I have the [▼] to a fair hearing.

I can [▼] a hearing by writing or [▼] the QHIP.

If you [▼] help for any family [▼], you will have to [▼] a different application form.

[▼], we will use the [▼] on this form to determine your [▼].

>>

Select Question(Link) ▼

SOCIO

The next questions will serve for statistical purposes only.

>>

Select Question(Link) ▼

Q33

What is your mother tongue?

- ◯ French
- ◯ English
- ◯ Other, please specify: ________

>>

Select Question(Link) ▼

Q34

What is your marital status?

- ◯ Single
- ◯ Married
- ◯ Common-law spouse
- ◯ Separated/Divorced
- ◯ Widow

>>

Select Question(Link) ▼

Q35

What is your employment status?

- ◯ Full-time employment (on preventive withdrawal or not, on maternity leave or not)
- ◯ Part-time employment (on preventive withdrawal or not, on maternity leave or not)
- ◯ Unemployed and looking for work
- ◯ Unemployed and NOT looking for work
- ◯ Student
- ◯ Retired
- ◯ Other, please specify: ________

>>

Select Question(Link) ▼

Q37

What is the size of your household?

- ◯ I live alone
- ◯ Including myself, our household has ____ people

>>

Select Question(Link) ▼

Q39

Have you ever been pregnant prior to this pregnancy (whether carried out to full term or not)?

- ◯ This is my 1st pregnancy
- ◯ This is my 2nd pregnancy
- ◯ This is my 3rd pregnancy
- ◯ This is at least my 4th pregnancy

>>

Select Question(Link) ▼

Q40

With which healthcare professional did you make the decision regarding whether or not to do the prenatal screening test for trisomy 21?

- ◯ Obstetrician-gynecologist
- ◯ Midwife
- ◯ Family physician
- ◯ I don't know
- ◯ I have never had to make that decision
- ◯ I made the decision alone
- ◯ Other, please specify: ________

>>

Select Question(Link) ▼

Q41

Which healthcare professional is monitoring your pregnancy:

- ◯ Obstetrician-gynecologist
- ◯ Midwife
- ◯ Family physician
- ◯ I don't know
- ◯ Other, please specify: ________

>>

Select Question(Link) ▼

Q42_3D

What was your weight BEFORE your pregnancy: ________ pounds (OR) ________ kg

What is your height: ____ feet ____ inches (OR) ____ metres ____ cm

>>

Select Question(Link) ▼

Q44_3D

Estimate the driving time between your home and the nearest hospital without traffic congestion, road works, road accidents, etc.

____ hour(s) ____ minute(s)

>>

Select Question(Link) ▾

Q45

Please write the first 3 characters of your zip code (example: G1L):

>>

Select Question(Link) ▾

MessComplete

Thank you, you have completed the survey.

Please click on the following arrow to receive your reward(s).

For any questions or comments, please contact Johanie Lépine, PhD. by email at: johanie.lepine@gmail.com, or by phone at: 418-525-4444 extension 53701.

>>
