# Supplementary material for: Role of Psychosocial Factors and Health Literacy in Pregnant Women’s Intention to Use a Decision Aid for Down Syndrome Screening: A Theory-Based Web Survey
Source: J Med Internet Res. 2016 Oct 28;18(10):e283. doi: 10.2196/jmir.6362 (PMC5106559; doi:10.2196/jmir.6362)
Supplement: Multimedia Appendix 2 [file jmir_v18i10e283_app2.pdf]

Decision Box
PATIENT
... to help you make a decision

UNIVERSITÉ LAVAL

Should I take the serum integrated test to screen for fetal Trisomy 21 (Down syndrome) ?

Who might consider being tested?

All pregnant woman. The risk of Trisomy 21 (T21) increases with the woman's age.

Number of women carrying a fetus with T21 according to age

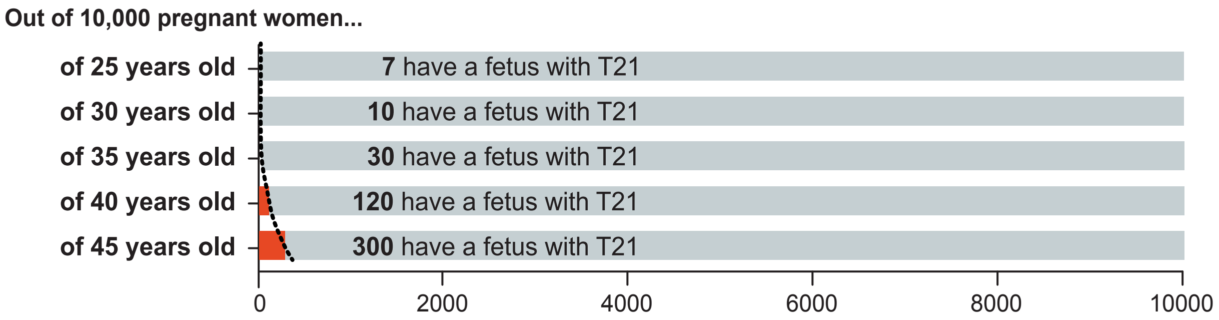

A decision to make

Doing and not doing the test are both acceptable choices. We suggest that you:

- base your decision on the best scientific evidence
- base your decision on your values and preferences
- share the decision with your health professional

Information to help you make the decision

What is the serum integrated test?

Two blood samples must be taken: **1st** : between 10 and 13 weeks of pregnancy
2nd : between 14 and 16 weeks of pregnancy

What is Trisomy 21 (or Down syndrome)?

- It is caused by having an extra copy of chromosome 21.
- People with T21 have slanted eyes, a thickened nuchal fold, a round face, poor muscle tone, very supple joints, greater risk of vision and hearing defects and cardiac and gastro-intestinal defects, and intellectual disabilities that vary from slight to moderate, but are sometimes serious.
- 60% of children with T21 require specialized home care.
- Some adults with T21 have jobs and are almost completely independent.
- People with T21 can develop meaningful emotional relationships and lead lives that are fulfilling for themselves and their families and friends.

What is the integrated serum test for?

- This test estimates the risk of carrying a fetus with T21.
- If the risk is high, the doctor offers an amniocentesis* to see if the fetus does have T21.
- This information helps you decide whether to prepare for a child with special needs or consider ending the pregnancy.

** What is an amniocentesis?*

An amniocentesis is a diagnostic test that checks the chromosomes of fetuses considered to be at risk of specific problems such as Down syndrome. A small sample of the liquid surrounding the fetus is taken using a needle inserted through the mother's abdomen into her uterus..

© Université Laval, 2014. All rights reserved

Doing or not doing the test

Doing the test

Benefits

Knowing your chances of carrying a fetus with T21

Out of each 10,000 women screened, 415 are identified as being at high risk of carrying a fetus with T21.

- If these women have an amniocentesis to verify the results of the screening, 15 would actually be carrying a fetus with T21.

Prepare to end the pregnancy

Out of 15 women who know they are carrying a fetus with T21, 10 choose to end the pregnancy.

Prepare for a child with T21

Out of 15 women who know they are carrying a fetus with T21, 5 choose to continue the pregnancy and can prepare for a child with T21.

Reassurance

Out of 10,000 women who take the test, 9585 are identified has at low risk of carrying a fetus with T21. These women are reassured.

Harms

False alarm

Of the 415 women identified as being at high risk of carrying a fetus with T21, 400 are not actually carrying a fetus with T21.

- many of these women experience anxiety.
- of these 415 women, 1 will have a miscarriage following an amniocentesis to verify test results.

Anxiety while waiting for results

Women waiting for test results (3 to 7 days) have anxiety levels 10 times higher than normal.

False reassurance

Two of the 9585 women identified as at low risk of carrying a fetus with T21 will actually be carrying a T21 fetus. These women will have been falsely reassured.

Not doing the test

Benefits

Avoid an unnecessary amniocentesis

Out of 10,000 women who take the test, 9983 are not carrying a fetus with T21. By not doing the test, 400 will avoid an unnecessary amniocentesis, and one woman will avoid a miscarriage caused by the amniocentesis.

Stay true to your personal convictions

For some women, not doing the test is in keeping with their personal convictions.

Avoid anxiety

Women who do not take the test avoid the anxiety of:

- waiting for the test results.
- making a decision about whether to do the amniocentesis or not if the test shows a high risk of T21.

Harms

Not knowing your risk of carrying a fetus with T21

Out of 10,000 women who do not take the test, 17 are carrying a fetus with T21. These women cannot prepare for:

- an increased chance of miscarriage: two of them will miscarry due to T21.
- living with a child with T21: 15 out of the 17 will carry the pregnancy to full term.

Anxiety due to the outcome of the pregnancy

Women who don't take the test may be anxious because they don't know if their child will have T21 or not.

▶ *Doing* *the test*

➤ Out of **10,000** pregnant women who do the test …

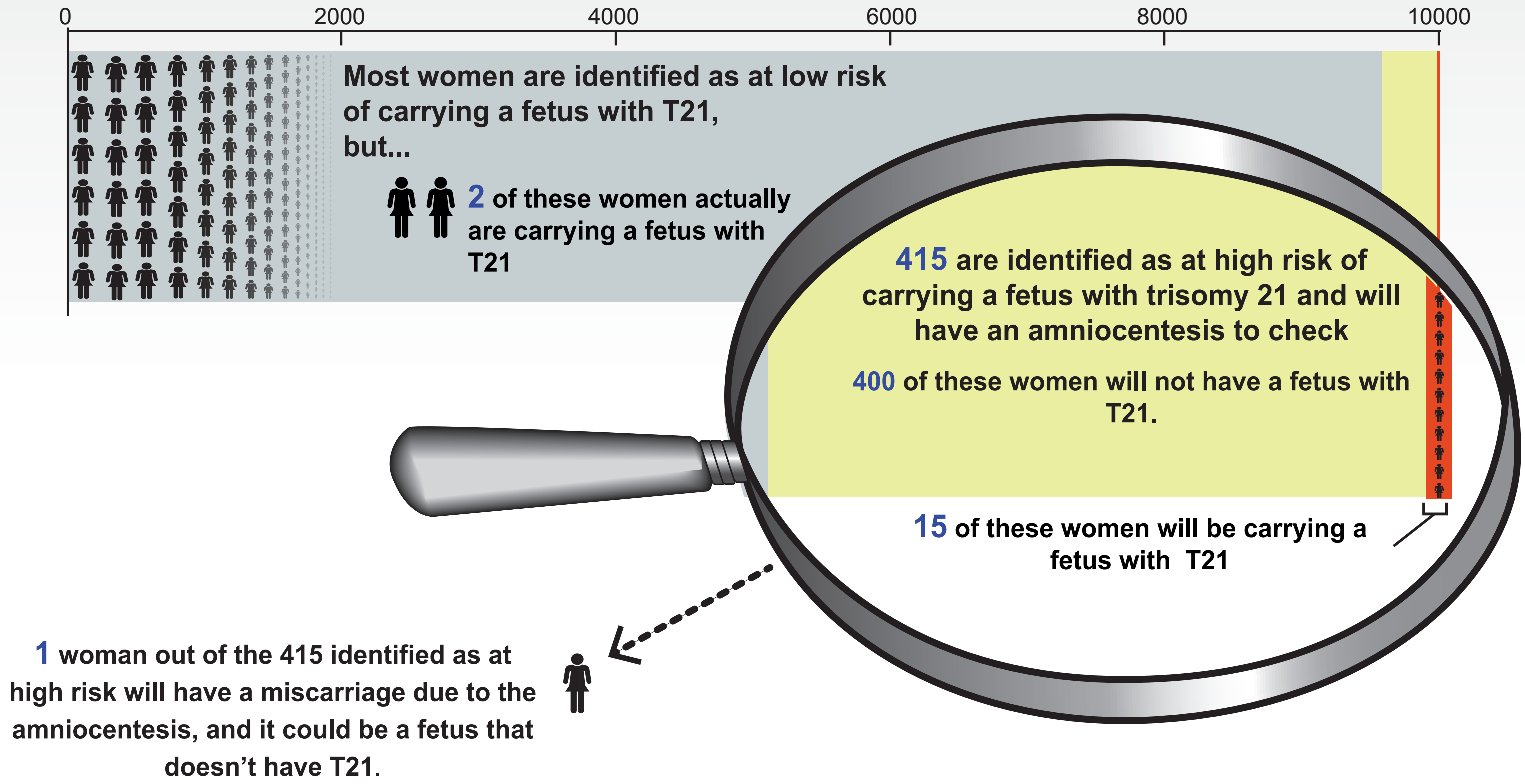

▶ *Not doing* *the test*

➤ Out of **10,000** pregnant women who do the test…

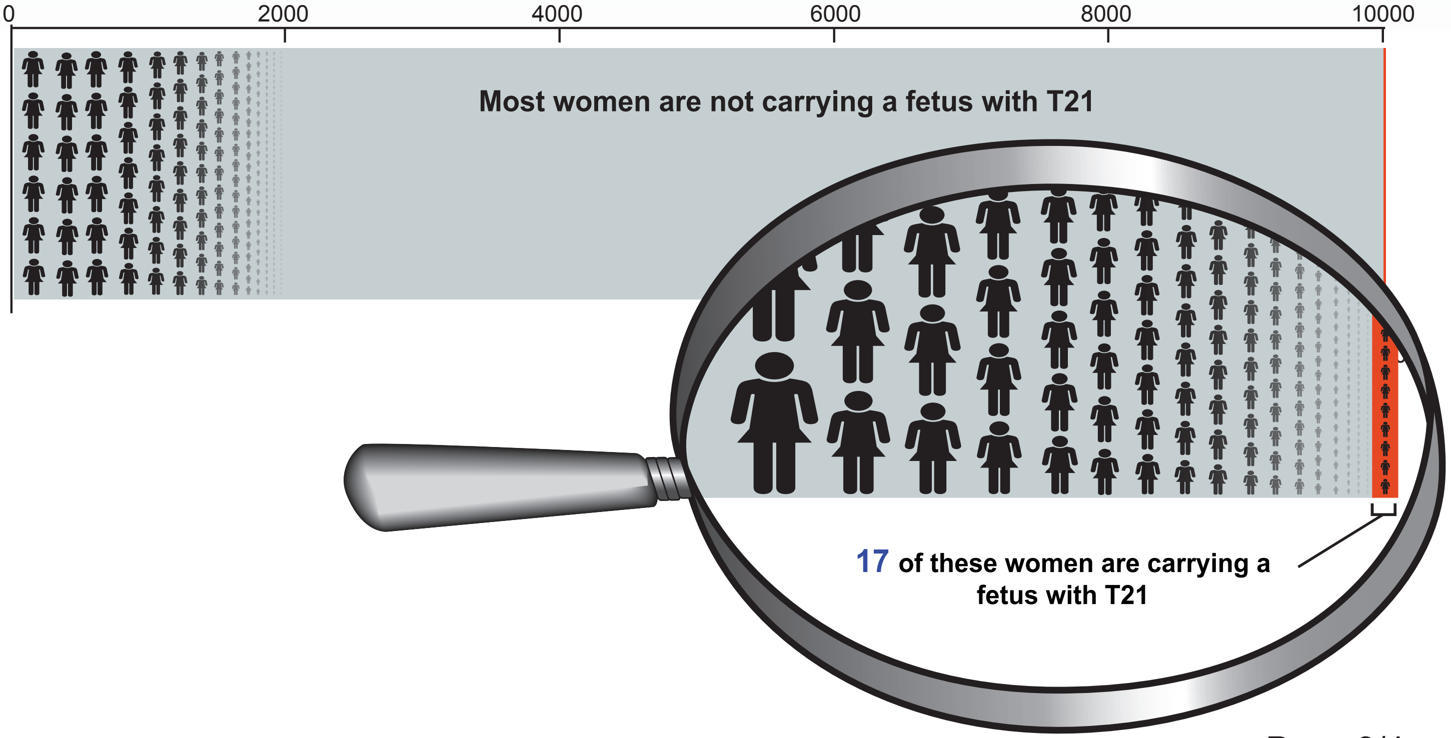

What are the benefits and harms that matter most to you?

Doing the test	Not doing the test
Benefits	***Benefits***
Harms	***Harms***
Other considerations:	***Other considerations:***

What is your decision?

Do the test	Don't do the test	I don't know
☐	☐	☐

Are you comfortable with this decision?

		Yes	No
**Sure of myself**	1) *Do you feel sure about the best choice for you?*	☐	☐
**Understand information**	2) *Do you know the benefits and harms of doing or not doing the test?*	☐	☐
**Risks/benefits ratio**	3) *Are you clear about which benefits and harms of doing or not doing the test matter most to you?*	☐	☐
**Encouragement**	4) *Do you have enough support and advice to make a choice?*	☐	☐

© SURE test; O'Connor & Légaré 2008

References

Schieve et. al. Disabil Health J. 2011; (4): 68–77. ACOG Practice Bulletin No. 77. Obstet Gynecol. Jan 2007;109(1): 217-227. Morris et al. J Med Screen. 2002; 9(1): 2-6. Malone et al. N Engl J Med. 2005; 353(19): 2001-2011. Wald et al. Health Technol Assess. 2003; 7(11): 1-77. Green et al. Health Technol Assess. 2004; 8(33): iii, ix-x, 1-109. Won et al. Prenatal diagnosis. 2005; 25(7): 608-611.

Authors: Anik Giguère (PhD), Maria-Esther Leiva-Portocarrero (BSc), Maria-Margarita Becerra-Perez (BSc), Hubert Robitaille (PhD) and France Légaré (MD, PhD), CHU de Québec Research Centre, Université Laval, Quebec, Canada.
No conflict of interest: This tool was developed with funding by a research grant from Genome Canada and Genome Quebec. Neither the granting agency, the authors, nor their affiliated organizations have interests at stake in the decisions made by patients after using this Decision Box.
Next update: March 2015.
