# Supplementary material for: Role of Psychosocial Factors and Health Literacy in Pregnant Women’s Intention to Use a Decision Aid for Down Syndrome Screening: A Theory-Based Web Survey
Source: J Med Internet Res. 2016 Oct 28;18(10):e283. doi: 10.2196/jmir.6362 (PMC5106559; doi:10.2196/jmir.6362)
Supplement: Multimedia Appendix 3 [file jmir_v18i10e283_app3.pdf]

Socio-cognitive variables scores and correlations (n=346)

Descriptive analyses			Spearman correlations[b]					
**Construct**	Mean[a] (SD)	Median[a] (Q1-Q3)	Att.	A.R.	S.N.	D.N.	M.N.	P.C.
Attitude (6 items)	3.86 (0.59)	3.83 (3.50-4.17)	1.00	0.47	0.68	0.55	0.60	0.46
Anticipated regret (2 items)	2.95 (1.04)	3.00 (2.00-4.00)	0.53	1.00	0.34	0.44	0.35	0.24
Subjective norm (3 items)	3.95 (1.04)	4.00 (3.33-4.33)	0.68	0.52	1.00	0.67	0.66	0.45
Descriptive norm (3 items)	3.79 (0.80)	4.00 (3.33-4.33)	0.55	0.44	0.67	1.00	0.56	0.43
Moral norm (3 items)	4.05 (0.87)	4.00 (3.67-5.00)	0.60	0.46	0.66	0.56	1.00	0.51
Perceived control (4 items)	4.23 (0.60)	4.25 (4.00-4.75)	0.46	0.15	0.45	0.43	0.51	1.00

Att.: Attitude; A.R.: Anticipated Regret; S.N.: Subjective Norm; D.N.: Descriptive Norm; M.N.: Moral Norm; P.C.: Perceived Control

[a]Out of 5

[b]$P < 0.0001$
