# Supplementary material for: Role of Psychosocial Factors and Health Literacy in Pregnant Women’s Intention to Use a Decision Aid for Down Syndrome Screening: A Theory-Based Web Survey
Source: J Med Internet Res. 2016 Oct 28;18(10):e283. doi: 10.2196/jmir.6362 (PMC5106559; doi:10.2196/jmir.6362)
Supplement: Multimedia Appendix 4 [file jmir_v18i10e283_app4.pdf]

Intention level by sociodemographic characteristics (n = 346)

Sociodemographic variable *n* (%)		Intention level[a] *n* (%)		
Ethnicity		<4	4	>4
White/Caucasian	319 (92.2)	99 (28.6)	86 (24.9)	134 (38.7)
Other	27 (7.8)	10 (2.9)	6 (1.7)	11 (3.2)
P				0.68 [b]
Education		<4	4	>4
No high school	4 (1.2)	1 (0.3)	1 (0.3)	2 (0.6)
High school diploma	25 (7.2)	11 (3.2)	6 (1.7)	8 (2.3)
Professional diploma	61 (17.6)	22 (6.4)	14 (4.1)	25 (7.2)
Collegial diploma	88 (25.4)	19 (5.5)	31 (9.0)	38 (11.0)
University degree	168 (48.6)	56 (16.2)	40 (11.6)	72 (20.8)
P				0.51 [b]
Annual family income		<4	4	>4
< $29 999	24 (6.9)	13 (3.7)	4 (1.2)	7 (2.0)
$30 000 - $59 999	74 (21.4)	22 (6.4)	22 (6.4)	30 (8.7)
$60 000 - $99 999	146 (42.2)	42 (12.1)	38 (11.0)	66 (19.1)
> $100 000	82 (23.7)	24 (6.9)	22 (6.4)	36 (10.4)
No answer	20 (5.8)	8 (2.3)	6 (1.7)	6 (1.7)
P				0.16 [b]
Mother tongue		<4	4	>4
French	318 (91.9)	101 (29.2)	87 (25.1)	130 (37.6)
English	18 (5.2)	5 (1.5)	4 (1.7)	9 (2.6)
Other	10 (2.9)	3 (0.9)	1 (0.3)	6 (1.7)
P				0.54 [b]
Civil status		<4	4	>4
Single	23 (6.7)	8 (2.3)	5 (1.5)	10 (2.9)
Married	88 (25.4)	29 (8.4)	19 (5.5)	40 (11.6)
Partner	235 (67.9)	72 (20.8)	68 (19.7)	95 (27.5)
P				0.95 [b]
Employment status		<4	4	>4
Unemployed	23 (6.7)	7 (2.0)	7 (2.0)	9 (2.6)
Part time	45 (13.0)	21 (6.1)	7 (2.0)	17 (4.9)
Full time	269 (77.7)	77 (22.3)	75 (21.7)	117 (33.8)
Student	9 (2.6)	4 (1.2)	3 (0.9)	2 (0.6)
P				0.22 [b]
Household		<4	4	>4
1 person	9 (2.6)	4 (1.2)	3 (0.9)	2 (0.6)
2 persons	142 (41.2)	42 (12.2)	41 (11.9)	59 (17.1)
3 persons	119 (34.5)	39 (11.3)	30 (8.7)	50 (14.5)
4 persons	55 (15.9)	16 (4.6)	13 (3.8)	26 (7.5)
5 persons	20 (5.8)	8 (2.3)	5 (1.5)	7 (2.0)
P				0.66 [b]
Pregnancy number		<4	4	>4
First	130 (37.6)	42 (12.1)	42 (12.1)	46 (13.3)
Second	137 (39.6)	48 (13.9)	32 (9.3)	57 (16.5)
Third	40 (11.5)	9 (2.6)	8 (2.3)	23 (6.7)
Fourth or more	39 (11.3)	10 (2.9)	10 (2.9)	19 (5.5)
P				0.12 [b]
Person helping to decide		<4	4	>4
Health professional	291 (84.1)	87 (25.1)	81 (23.4)	123 (35.6)

Alone	49 (14.2)	19 (5.5)	10 (2.9)	20 (5.8)	
Don't know	6 (1.7)	3 (0.9)	1 (0.3)	2 (0.6)	
P					0.53 [b]
Monitored by		<4	4	>4	
Family physician	105 (30.3)	38 (11.0)	27 (7.8)	40 (11.6)	
Obstetrician-gynecologist	201 (58.1)	58 (16.8)	52 (15.0)	91 (26.3)	
Midwife	30 (8.7)	9 (2.6)	11 (3.2)	10 (2.9)	
Other	10 (2.9)	4 (1.2)	2 (0.6)	4 (1.2)	
P					0.49 [b]

[a] Score range from 1 (-) to 5 (+)

[b] Bivariate ordinal logistic regression
