# Supplementary material for: Role of Psychosocial Factors and Health Literacy in Pregnant Women’s Intention to Use a Decision Aid for Down Syndrome Screening: A Theory-Based Web Survey
Source: J Med Internet Res. 2016 Oct 28;18(10):e283. doi: 10.2196/jmir.6362 (PMC5106559; doi:10.2196/jmir.6362)
Supplement: Multimedia Appendix 5 [file jmir_v18i10e283_app5.pdf]

Bivariate analysis of health literacy, by intention category (n = 346)

	Health literacy *n* (%)	Intention level[a] *n* (%)			
Objective health literacy S – TOFHLA[b]		<4	4	>4	
Median (Q1-Q3)	N/A	36.00 (34.00-36.00)	36.00 (35.00-36.00)	36.00 (35.00-36.00)	
P					0.27[c]
Subjective health literacy – 3HLQ[d]		<4	4	>4	
Inadequate (<10)	168 (45.55)	51 (14.74)	53 (15.32)	64 (18.50)	
Adequate (≥10)	178 (51.45)	58 (16.76)	39 (11.27)	81 (23.41)	
P					0.52[3]
Objective numeracy– 3NQ[e]		<4	4	>4	
One error or more	150 (43.35)	46 (13.29)	34 (9.83)	70 (20.23)	
All correct answers	196 (56.65)	63 (18.21)	58 (16.76)	75 (21.68)	
P					0.26[3]
Subjective numeracy– SNS-total[f]		<4	4	>4	
< median (3.88/5)	155 (44.80)	53 (15.32)	40 (11.56)	62 (17.92)	
≥ median (3.88/5)	191 (55.20)	56 (16.18)	52 (15.03)	83 (23.99)	
P					0.37[3]
Subjective numeracy– SNS-cognitive[f]		<4	4	>4	
< median (3.75/5)	156 (45.09)	49 (14.16)	38 (10.98)	69 (19.94)	
≥ median (3.75/5)	190 (54.91)	60 (17.34)	54 (15.61)	76 (21.97)	
P					0.61[3]
Subjective numeracy– SNS-preference[f]		<4	4	>4	
< median (4.00/5)	141 (40.75)	45 (13.01)	45 (13.01)	51 (40.75)	
≥ median (4.00/5)	205 (59.25)	64 (18.50)	47 (13.58)	94 (27.17)	
P					0.24[3]

[a] Score range from 1 (-) to 5 (+)
[b] Score range from 0 (-) to 36 (+)
[c] Bivariate ordinal logistic regression
[d] Score range from 0 (-) to 12 (+)
[e] Score range from 0 (-) to 3 (+)
[f] Score range from 1 (-) to 5 (+)
N/A: Not Applicable
