# Supplementary material for: Role of Psychosocial Factors and Health Literacy in Pregnant Women’s Intention to Use a Decision Aid for Down Syndrome Screening: A Theory-Based Web Survey
Source: J Med Internet Res. 2016 Oct 28;18(10):e283. doi: 10.2196/jmir.6362 (PMC5106559; doi:10.2196/jmir.6362)
Supplement: Multimedia Appendix 6 [file jmir_v18i10e283_app6.pdf]

	Descriptive analyses				Spearman Correlations *P*					
	Range (-/+)	Mean (SD)	Median (Q1-Q3)	α[a]	Att.	A.R.	S.N.	D.N.	M.N.	P.C.
**Objective health literacy**										
S-TOFHLA	0-36	33.52	36.00	0.96	0.003	-0.01	0.05	0.13	0.14	0.24
		(5.79)	(35.00-36.00)		.96	.81	.40	.01	.008	<.0001
Subjective health literacy										
3HLQ[1]	0-12	9.40	10.00	0.70	0.07	-0.15	0.04	0.05	0.16	0.27
		(1.97)	(8.00-11.00)		.22	.004	.45	.39	.002	<.0001
**Objective numeracy**										
3NQ	0-3	2.40	3.00	0.51[b]	-0.13	-0.13	-0.01	-0.02	0.03	0.10
		(0.80)	(2.00-3.00)		.01	.02	.86	.76	.57	.07
**Subjective numeracy**										
SNS	1-5	3.82	3.88	0.79	0.09	-0.08	0.05	0.06	0.08	0.25
– total		(0.71)	(3.38- 4.38)		.11	.13	.32	.24	.13	<.0001
SNS	1-5	3.99	4.00	0.51	0.18	-0.02	0.18	0.18	0.20	0.28
– preference		(0.68)	(3.50-4.50)		.001	.68	.001	.001	.0002	<.0001
SNS	1-5	3.65	3.75	0.89	-0.02	-0.10	-0.05	-0.04	-0.02	0.15
– cognitive		(1.00)	(3.50-4.50)		.73	.05	.36	.49	.70	.0049

Associations between health literacy scores and sociocognitive factors (*n*=346)

Att.: Attitude; A.R.: Anticipated Regret; S.N.: Subjective Norm; D.N.: Descriptive Norm; M.N.: Moral Norm; P.C.: Perceived Control

[a] Coefficient Cronbach Alpha

[a] Scoring for this scale has been reversed from its original position (Chew et. al) [80] so that health literacy ranges for all scales are easier to compare.

[b] KR20
