# Supplementary material for: Role of Psychosocial Factors and Health Literacy in Pregnant Women’s Intention to Use a Decision Aid for Down Syndrome Screening: A Theory-Based Web Survey
Source: J Med Internet Res. 2016 Oct 28;18(10):e283. doi: 10.2196/jmir.6362 (PMC5106559; doi:10.2196/jmir.6362)
Supplement: Multimedia Appendix 7 [file jmir_v18i10e283_app7.pdf]

Health literacy measure correlations (*n*=346)

Spearman correlations, *n* = 346
P

	Health literacy		Numeracy			
	Objective	Subjective	Objective	Subjective		
				total	cognitive	preferences
**Health literacy**						
Objective (S-TOFHLA[a])	1.00	0.17	0.25	0.18	0.17	0.14
		.001	<.0001	.001	.001	.001
Subjective (3HLQ[b])	0.17	1.00	0.18	0.31	0.25	0.25
	.001		.001	<.0001	<.0001	<.0001
**Numeracy**						
Objective (3NQ[c])	0.25	0.18	1.00	0.19	0.19	0.14
	<.0001	.001		.0003	.0005	.007
Subjective (SNS[d])						
SNS – total	0.18	0.31	0.19	1.00	0.89	0.71
	.001	<.0001	.0003		<.0001	<.0001
SNS – cognitive	0.17	0.25	0.19	0.89	1.00	0.33
	.001	<.0001	.0005	<.0001		<.0001
SNS – preferences	0.14	0.25	0.14	0.71	0.33	1.00
	.001	<.0001	.007	<.0001	<.0001	

[a]Range from 1 (-) to 36 (+)
[b]Range from 1 (-) to 12 (+)
[c]Range from 1 (-) to 3 (+)
[d]Range from 1 (-) to 5 (+)
